# Right Paraduodenal Hernia: A Rare Cause of Small Bowel Strangulation

**DOI:** 10.7759/cureus.11807

**Published:** 2020-11-30

**Authors:** Aishwarya Reddy Bollampally, Baskaran Dhanapal, Faiz Hussain Mohammed

**Affiliations:** 1 Surgery, Apollo Institute of Medical Sciences and Research, Hyderabad, IND

**Keywords:** internal hernia, intestinal obstruction, paraduodenal hernia

## Abstract

Internal hernias are a rare cause of intestinal obstruction. Among the internal hernias, left paraduodenal hernia is the most typical type followed by the right paraduodenal hernia. It is impossible to make a clinical diagnosis of internal hernia, as there are no specific symptoms or physical signs. A high degree of suspicion is required, and an accurate diagnosis can be made using cross-sectional imaging of the abdomen like a Computed Tomography (CT) scan or Magnetic Resonance Imaging (MRI) scan. In this case report, we present our experience in managing a patient who had a right paraduodenal hernia with small bowel strangulation. We present this case report to highlight the importance of considering internal hernias like right *paraduodenal* hernia in the differential diagnosis of intestinal obstruction.

## Introduction

Internal hernia is an abnormal protrusion of the abdominal viscera through a congenital or acquired defect in the peritoneum or mesentery. Internal hernias are a rare cause of intestinal obstruction. Acquired internal hernias usually occur following surgeries like Roux-en-Y gastric bypass (RYGB). In Congenital type, the herniation may either occur through normal foramina or recesses like the foramen of Winslow or through abnormal defects that arise due to disorder in the bowel rotation and fixation. Types of internal hernia include: Left paraduodenal hernia; Right paraduodenal hernia; Transmesenteric hernia; Foramen of Winslow hernia; Pericaecal hernia; Transomental hernia; Sigmoid mesocolon related hernia; Supravesical hernia [1]. The left paraduodenal fossa (Fossa of Landzert) hernia is the most common type, followed by the right paraduodenal fossa (fossa of Waldeyer) hernia [2].

The right paraduodenal hernia occurs due to abnormality in the second stage of embryonic midgut rotation. Although congenital, most patients are entirely asymptomatic and diagnosed incidentally during abdominal surgery or abdominal imaging [2]. The clinical presentation in symptomatic patients can vary from recurrent episodes of abdominal pain to acute intestinal obstruction or strangulation [2]. It is impossible to make a clinical diagnosis of right paraduodenal hernia as there are no specific symptoms or signs. Small bowel contrast studies may be helpful, but a Computed Tomography (CT) scan with oral and intravenous contrast is the best modality to establish the diagnosis [1,2]. Although right paraduodenal hernia accounts for only 0.2 to 0.9% of all intestinal obstruction cases, the morbidity and mortality are high in patients with an acute presentation [3]. In this case report, we present our experience in managing a patient of right paraduodenal hernia with small bowel strangulation.

## Case presentation

A 29-year-old male patient presented to our casualty with sudden onset diffuse abdominal pain, abdominal distension, and bilious vomiting. He had no history of previous abdominal surgery. He had few episodes of abdominal pain and vomiting in the past, which resolved with conservative management. He had no known comorbid illnesses. He underwent an ultrasound scan of the abdomen during one of the episodes, which was inconclusive.

**Figure 1:**
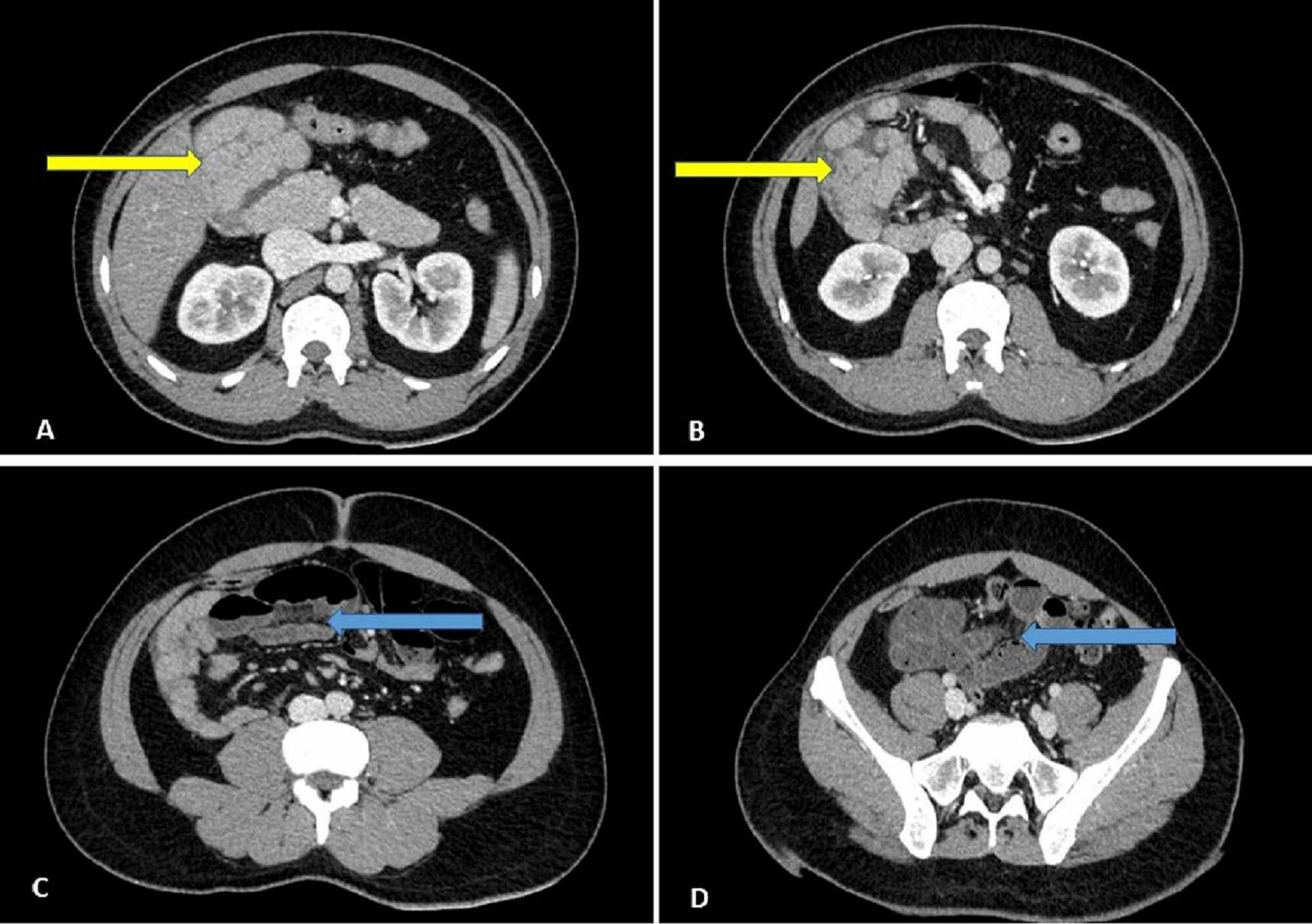
Coronal sections of the contrast-enhanced CT scan of the abdomen demonstrating a cluster of small bowel loops in the right side of the abdomen (yellow arrows). The ischemic bowel loops (blue arrows). CT scan - Computed Tomography scan

At presentation, he was dehydrated and tachycardic but not hypotensive. Abdominal examination revealed diffuse tenderness and guarding. Blood investigations revealed a leukocytosis. The serum amylase, lipase, renal and liver function tests were within normal limits. A CT scan of the abdomen with intravenous contrast was done, which showed a cluster of small bowel loops present to the right side of the duodenum, and there was no contrast enhancement in some small bowel loops (Figure 1).

A diagnosis of right paraduodenal hernia with small bowel strangulation was made, and an immediate laparotomy was performed. During surgery, we noted that the small bowel loops were ischemic. The proximal jejunum was seen entering into a hernia sac located posterior to the ascending and transverse colon. The caecum and ascending colon were mobilized, the sac was opened on the lateral aspect near the ascending colon, and the small bowel loops were reduced. The small and large bowel were returned to their regular anatomical positions. After reducing the bowel to their anatomical position and applying warm laparotomy pads, the bowel loops became viable, and no bowel resection was required. He had an uneventful postoperative recovery. At a review visit in the outpatient department four weeks after surgery, he had no complications.

## Discussion

Paraduodenal hernias are the most common type of internal hernia, accounting for approximately 53% of internal hernia cases [2,3]. Left paraduodenal hernia is the most common type [2]. Right paraduodenal hernia is a congenital disorder that occurs due to abnormality in the midgut rotation and fixation. The primitive midgut, which lies outside the abdominal cavity, is divided into pre-arterial and post-arterial segments by the superior mesenteric artery as its axis. The pre-arterial segment forms the distal duodenum, jejunum and proximal ileum. The distal ileum, caecum, ascending colon, and proximal transverse colon develops from the post-arterial segment. As the midgut loops return into the abdominal cavity, they undergo a 270° counterclockwise rotation. The pre-arterial segment returns first, followed by the post-arterial segment. In the Right paraduodenal hernia, the pre-arterial segment arrests in the second stage of rotation and continues to remain in the right side of the abdominal cavity. The post-arterial segment rotates usually, and its mesentery fuses to the posterior abdominal wall, entrapping the pre-arterial bowel loops in a peritoneal sac [2,3]. The superior mesenteric artery, middle colic artery, and ileocolic artery lies on the sac's anterior surface, on the medial aspect [3].

Right paraduodenal hernia is more common in males and mostly presents in the fourth to sixth decade. The earliest age of presentation documented in published literature is in a 1-week old neonate [4]. They are mostly asymptomatic and may be diagnosed incidentally during surgery. Symptoms can vary from recurrent episodes of abdominal pain and vomiting to intestinal obstruction. A CT scan is the best method to establish the diagnosis [2]. A high degree of suspicion is required while interpreting the CT images. In a case of right paraduodenal hernia, the CT scan will show a cluster of small bowel loops to the right of the duodenum, behind the superior mesenteric vessels [5]. Sometimes the trapped bowel loops can be mistaken for a mass lesion prompting a biopsy, which can cause catastrophic complications. Hence it is essential to perform a CT scan after administering oral contrast, especially in patients who do not have clinical findings suggestive of intestinal obstruction.

Surgical intervention is required if the diagnosis is established conclusively, even in asymptomatic patients because of the high risk of intestinal obstruction and strangulation. There is a 50% risk of bowel incarceration in the untreated paraduodenal hernia [1,2]. Both open and laparoscopic approaches have been described [5]. Care should be taken while opening the sac's neck to avoid injury to the superior mesenteric vessels and its branches. The morbidity and mortality are high in patients who require bowel resection. With the increased availability of CT scans, a more significant number of asymptomatic patients may be diagnosed incidentally.

## Conclusions

To conclude, a CT scan should be considered in evaluating a patient presenting with recurrent episodes of abdominal pain. There should be a high degree of suspicion for an internal hernia while interpreting the CT images of such patients. Elective surgery, preferably by laparoscopic approach, is recommended to prevent the occurrence of complications, even in incidentally diagnosed patients.
